# Longitudinal kinetics of the viral infection biomarker 3′-deoxy-3′,4′-didehydro-cytidine in SARS-CoV-2, influenza A virus and RSV human challenge models

**DOI:** 10.1038/s44298-025-00132-x

**Published:** 2025-06-20

**Authors:** Ravi Mehta, Elena Chekmeneva, Stephanie Ascough, Helen Wagstaffe, Loukas Papargyris, Malick Gibani, Ada H. Y. Yuen, Maria Valdivia-Garcia, Caroline Sands, María Gómez-Romero, Ewurabena A. Mills, Vincenzo Sgro, Lydia Slater, Pete Dayananda, Claire Broderick, Jiayun Xu, Myrsini Kaforou, Lynn Maslen, Mahdad Noursadeghi, Andrew J. Pollard, Wendy Barclay, Zoltan Takats, Christopher Chiu, Shiranee Sriskandan

**Affiliations:** 1 Department of Infectious Disease, Imperial College London, London, UK; 2 National Phenome Centre, Section of Bioanalytical Chemistry, Department of Metabolism, Digestion and Reproduction, Imperial College London, Hammersmith Hospital Campus, IRDB Building, London, UK; 3 Division of Infection and Immunity, University College London, London, UK; 4 Oxford Vaccine Group, Department of Paediatrics, and the NIHR Oxford Biomedical Research Centre, University of Oxford, Oxford, UK; 5 NIHR Health Protection Research Unit in Healthcare-associated Infection & Antimicrobial Resistance, Imperial College London, London, UK; 6 Centre for Bacterial Resistance Biology, Imperial College London, London, UK

**Keywords:** Medical research, Virology

## Abstract

3’-deoxy-3’,4’-didehydro-cytidine (ddhC) is a recently discovered host biomarker for viral infections, though its temporal kinetics remain unclear. This study tests the hypothesis that ddhC is an acute phase reactant, rising shortly after viral infection and subsequently falling to baseline. We leveraged the precise monitoring facilitated by human challenge studies to investigate healthy participants inoculated with SARS-CoV-2, influenza A virus (H3N2), or respiratory syncytial virus (RSV). Using targeted liquid chromatography-tandem mass spectrometry, we quantified ddhC concentrations in serial plasma samples collected pre- and post-inoculation. In SARS-CoV-2 and H3N2 influenza A virus infection, but not RSV, ddhC levels peaked at 3–7 days post inoculation and declined to baseline by days 10–14. This pattern was also observed in asymptomatic or paucisymptomatic participants. A comparison of ddhC concentrations with matched timepoint whole blood gene expression revealed a correlation with interferon-related genes, including viperin and CMPK2—enzymes implicated in its upstream biosynthesis. Our results suggest that ddhC is a biomarker of the acute phase of viral infection, with potential to guide early interventions that reduce antimicrobial resistance and strengthen pandemic preparedness. Future work should explore ddhC dynamics in natural and experimental infections across varying severities and assess its utility in diverse populations and healthcare settings.

## Introduction

3′-deoxy-3′,4′-didehydro-cytidine (ddhC) is the free base of ddhC-triphosphate (ddhCTP), an antiviral small molecule encoded by the human genome^1^. We previously demonstrated that ddhC acts as an accurate biomarker for multiple viral infections by analysing sera from patients hospitalised with a range of viral infections—including SARS-CoV-2, influenza A virus, respiratory syncytial virus (RSV), measles, and dengue—and comparing them to patients with bacterial infections and those without infection^2^. Other groups have confirmed significantly elevated ddhC levels in patients with SARS-CoV-2 infection^3,4^. A pan-viral biomarker such as ddhC has several key applications, including reducing antibiotic overuse by differentiation of viral from bacterial infections, thereby combating antibiotic resistance, and in pandemic preparedness by enabling rapid identification of viral infections when pathogen-specific diagnostics are unavailable. However, translating ddhC into clinical use requires an understanding of temporal changes in its concentration during a viral infection. For instance, its clinical utility would differ markedly depending on whether ddhC remains elevated for weeks after infection, or instead rises briefly for only a few hours or days. Therefore, we sought to test the hypothesis that ddhC acts as a virally induced acute-phase reactant, rising in the days following viral infection and subsequently falling back to baseline as the infection resolves.

Human challenge studies provide a controlled method to test this type of hypothesis. Deliberate, controlled infection of healthy volunteers, with extensive post-infection monitoring, enables longitudinal examination of the immune response over time, with unique access to pre-infection baseline samples and specimens collected during the asymptomatic incubation period. In this pilot study, we investigated ddhC concentrations in sequential plasma samples taken from healthy volunteers who underwent challenge with one of: SARS-CoV-2^5^, H3N2 influenza A virus^6^ or RSV^7^. We compared ddhC concentrations in participants who were infected and symptomatic, infected and asymptomatic or paucisymptomatic, and those who did not develop infection post-challenge. Samples from participants challenged with *Salmonella* Typhi were used as bacterial infection controls^8^.

The production of ddhCTP, and consequently ddhC, is thought to be dependent on two interferon-stimulated genes, viperin and CMPK2, which catalyse the conversion of CTP to ddhCTP through a radical S-adenosylmethionine (SAM) pathway^1^. In this study, we also investigated the relationship between viperin and CMPK2 gene expression and ddhC concentration over time. Our results provide the first insights into the kinetics of the human ddhC response to viral infection.

## Methods

### Study design and samples

We sourced samples from three independent viral human challenge studies conducted in the United Kingdom. Study methodologies have been described in detail elsewhere^5–7^. Briefly, healthy volunteers aged 18-30 years (SARS-CoV-2) or 18-55 years (influenza A virus & RSV) were screened and recruited for intranasal challenge with the respective virus, followed by quarantine in a clinical research facility. Daily recording of symptoms, measurement of nasal viral loads (via lavage for influenza A virus and RSV, flocked swabs for SARS-CoV-2), and collection of plasma samples were undertaken. Post hoc, a small subset of samples from an older cohort of participants (aged 60-75 years) from the same RSV challenge study was also investigated.

**Fig. 1:**
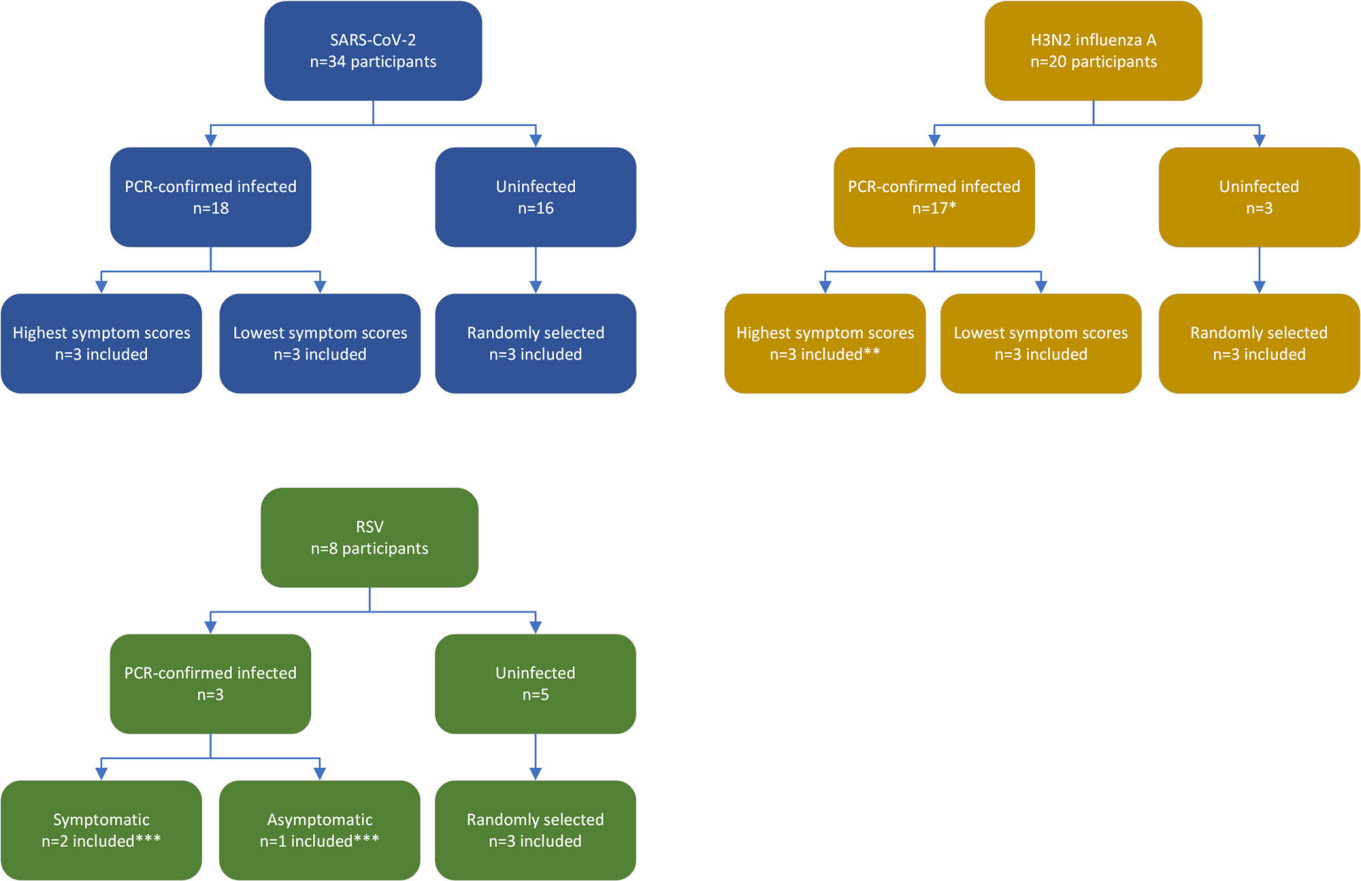
Study overview. For SARS-CoV-2 and influenza A virus challenge studies, the three most and three least symptomatic participants who had PCR-confirmed infection were included, based on symptom scores. For RSV, all three PCR-confirmed infected participants were included, two of whom were symptomatic. *In the original challenge study, one participant was found co-infected with rhinovirus and so excluded from this study. **Participants ranking 1st, 3rd and 4th in terms of highest symptom score were selected, as not all samples for the participant ranking 2nd were available. ***Symptomatic/asymptomatic status was defined as per the original study.

From the SARS-CoV-2 and influenza A virus challenge studies, we randomly selected three participants who did not develop infection; three who developed infection (confirmed by polymerase chain reaction [PCR]; one infected influenza A participant was excluded due to concomitant detection of rhinovirus by PCR) and were most symptomatic based on highest symptom scores; and three who developed PCR-confirmed infection but were paucisymptomatic or asymptomatic based on lowest symptom scores (Fig. 1, see Supplementary Table 1 for scores). The RSV challenge study resulted in three PCR-confirmed infected participants, two of whom were symptomatic as per study definitions^7^. For all challenge studies, uninfected participants were randomly chosen using a random number generator in Excel v16.9. For each participant, plasma samples pre-virus inoculation and from days 1, 3, 5, 7, 10, 14, and (influenza A virus and RSV only) 28 post-virus inoculation underwent ddhC measurement. All samples were retrieved from long-term storage at −80 °C. The subset of plasma samples from three infected participants in the older RSV cohort was from pre-inoculation, day 3 and day 6 post inoculation.

As a control group, we tested plasma samples from ten participants from a *Salmonella* Typhi human challenge study that was conducted independently prior to this study and is described in detail elsewhere^8^. Briefly, 21 healthy volunteers underwent oral challenge with wild-type *S*. Typhi, from which 15 participants met the composite diagnostic end-point of *S*. Typhi bacteraemia and/or fever ≥38 °C for ≥12 h. The ten participants selected as controls all reached the bacteraemic end-point and had at least one day 0 sample and one sample from the day of *S*. Typhi bacteraemia. Additional samples from day 2, 4 and 28 post-challenge samples were included where available.

### ddhC measurement

To accurately measure ddhC concentrations, we developed and fully validated a targeted ddhC assay using liquid chromatography coupled with tandem mass spectrometry (LC-MS/MS). The ddhC analytical standard and ^13^C_5_-ddhC stable isotope labelled internal standard (SIL-IS) were custom synthesised by High Force Research™ (https://highforceresearch.com/). The ddhC analytical standard was also purchased from Berry and Associates (cat. no. PY 7790). Detailed information regarding other chemicals and materials, method validation, clinical samples used in validation, the surrogate and zero (analyte-free) matrix used for calibration and quality control, and data processing is described in the Supplementary.

The LC instrument setup consisted of a Waters Acquity UPLC solvent management system and a Waters 2777C external autosampler (Waters, Wilmslow, UK). Chromatographic separation was performed on a Waters BEH HILIC 2.1 × 100 mm, 1.7 μm column (Waters, Wilmslow, U.K.). Mobile phase A consisted of 20 mM ammonium formate with 0.1% formic acid in water (v/v), and mobile phase B was 0.1% formic acid in acetonitrile (v/v). The weak and the strong washes were 1:3 water/acetonitrile (v/v) and 100% isopropanol, respectively. To avoid carryover observed during the method development, the needle wash cycle in the autosampler method was extended to three washes prior to and after the injection.

The chromatographic column was maintained at 55 °C during the run, and the LC gradient was performed at 0.8 mL/min starting at 3% A for 0.1 min followed by an increase to 20% A at 1.5 min, which was maintained for the next 0.25 min. This was followed by an increase to 50% A at 2 min and maintained until 3.5 min to elute all sample material from the column. At 3.6 min, it was returned to the initial LC conditions of 3% A for re-equilibration, ending at 7.5 min.

MS detection was performed with a Waters Xevo TQ-S tandem quadrupole instrument (Waters, Wilmslow, UK) using electrospray ionisation (ESI) in positive ion mode. Multiple reaction monitoring (MRM) was used for the quantification of ddhC; the specific metabolite and labelled standard MRM transitions are presented in Supplementary Table 4. The cone voltage for all transitions was of 4 V. Nitrogen was used as desolvation gas, and argon was used as collision gas. The following source conditions were used for the run: capillary voltage of 2.5 kV; source offset of 30 V; desolvation temperature of 600 °C; source temperature of 150 °C, desolvation gas flow of 1200 L/h; cone gas flow 250 L/h; nebuliser gas of 7.0 bar; collision gas of 0.18 mL/min.

For ddhC quantification, study plasma samples stored at −80 °C were thawed overnight at 4 °C and then vortex-mixed. Aliquots of 40 μL of each sample were added to 96-deep-well polypropylene plates (2 mL, Eppendorf). Subsequently, samples were diluted 1:1 with LC-MS grade water, and 16 μL of 600 ng/mL aqueous solution of ^13^C_5_-ddhC SIL-IS were spiked into each sample. Three parts of ice-cold acetonitrile (288 μL) were then added to one part (96 μL) of diluted sample for protein precipitation. Each plate was sealed prior to mixing at 1400 rpm for 2 h at 4 °C (MixMate, Eppendorf). The plates were then centrifuged for ten minutes at 3486 × *g* and 4 °C, and the supernatants (125 µL) were transferred into 96-well polypropylene plates (Eppendorf), which were heat sealed and centrifuged for five minutes at 3486 × *g* and 4 °C prior to the LC-MS/MS analysis.

Preparation of the calibration solutions and QC samples followed the same protocol, starting with aliquots of 40 μL of each concentration level working solution in LC-MS water and were diluted 1:1 with surrogate matrix of 2% BSA in PBS. The applicability of surrogate matrix for ddhC analysis was assessed in a parallelism study detailed in the Supplementary. The full description of study and QC samples preparation, sample formatting and run order is given in the Supplementary.

### RNA sequencing and correlation with ddhC

For all participants in the SARS-CoV-2 and influenza A virus challenge studies, blood samples were taken at sequential timepoints for RNA sequencing (days 0, 1, 2, 3, 4, 5, 7, 10, 14, and 28 for SARS-CoV-2, and days 0, 1, 2, 3, 7, 10, 14 and 28 for influenza A virus). RNA sequencing methodology for both studies has been previously described^9^. RNA sequencing was not performed for the RSV challenge study. Gene counts were normalised using transcripts per million normalisation for SARS-CoV-2, and using the DESeq2 package in R^10^ for influenza A virus. The relationship between log_2_-transformed normalised gene counts and log_2_-transformed ddhC concentrations was assessed using Pearson correlation.

### Statistics

Analysis was done in Excel v16.9, GraphPad Prism and R^11^. Mean ddhC concentrations and standard errors were plotted using GraphPad Prism. An unpaired two-tailed *t*-test was used to compare peak ddhC levels between symptomatic and asymptomatic/paucisymptomatic groups and between viral challenge and typhoid challenge groups. For RNA sequencing data analysis, Pearson correlation coefficients and *p*-values were calculated using the cor and cor. Test packages in R with default parameters.

### Ethics

Ethical approval to sample participants was granted for the original human challenge studies (reference numbers: 20/UK/0002 [SARS-CoV-2 challenge], 19/LO/1441 [H3N2 influenza A challenge], 11/LO/1826 [RSV challenge], A16/SC/0358 [typhoid challenge])^5–8^. Ethical approval was separately granted to use anonymised, stored samples from previously conducted human studies to investigate the response to infection (reference 06/Q0406/20).

Approval to use consented healthy donor plasma samples from a sub-collection of the Imperial College Healthcare Tissue Bank for infection and biomarker research was also granted (reference 12/WA/0196, project R12023).

## Results

### ddhC is a viral acute-phase reactant in SARS-CoV-2 and influenza A virus challenge

Sequential plasma samples from nine SARS-CoV-2, nine H3N2 influenza A virus and six RSV challenge participants (Supplementary Table 2) underwent ddhC measurement using targeted LC-MS/MS.

**Fig. 2:**
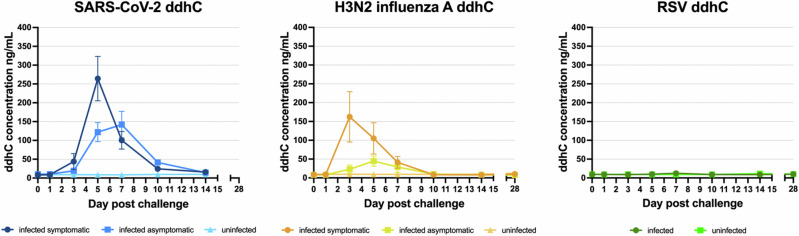
The ddhC response to human challenge with SARS-CoV-2 and influenza A virus, but not RSV, follows an acute phase reactant pattern over time. Results are expressed as mean ± s.e.m. for the three participants per category (infected symptomatic vs infected asymptomatic vs uninfected for SARS-CoV-2 and influenza A virus; infected vs uninfected for RSV). ‘Asymptomatic’ includes both asymptomatic and paucisymptomatic participants. Day 28 samples for SARS-CoV-2 were unavailable.

A clear ddhC response was detected in human challenge participants who developed PCR-confirmed infection following challenge with SARS-CoV-2 and influenza A virus, but not RSV (Fig. 2). ddhC concentrations remained under 20 ng/mL in all participants who did not develop infection post challenge. The ddhC response in all patients infected with SARS-CoV-2 and influenza A virus followed an acute phase reactant pattern, rising to a maximum between day 3 and 7 post viral inoculation, and falling to baseline between day 10 and 14 (Fig. 2). In SARS-CoV-2 and influenza A virus infections, ddhC was elevated in both symptomatic and paucisymptomatic or asymptomatic participants. Symptomatic participants trended towards a higher mean peak ddhC concentration compared with asymptomatic participants, although this difference was not statistically significant (264 ng/mL vs. 142 ng/mL in SARS-CoV-2, *p* = 0.15; 162 ng/mL vs 45 ng/mL in influenza A virus, *p* = 0.10). In contrast, the mean peak ddhC concentration in infected RSV participants was low, similar to uninfected participants (13 ng/mL vs. 12 ng/mL). A similar observation was noted when ddhC concentrations were measured in a limited subset of samples from older participants (aged 60–75 years) in a separate arm of the same RSV challenge study, with the mean peak ddhC concentration remaining comparable to baseline at 18 ng/mL (Supplementary Fig. 1).

ddhC concentrations in *S*. Typhi peaked after challenge on the day of bacteraemia, which occurred between 4 and 13 days post-challenge among participants. The mean peak concentration was 30 ng/mL (range 14–48 ng/mL, Supplementary Fig. 2), which was significantly lower than the mean peak ddhC concentrations observed in symptomatic and asymptomatic SARS-CoV-2 (264 ng/mL and 203 ng/mL, *p* < 0.001) and symptomatic influenza A virus (162 ng/mL, *p* < 0.001), but not significantly different to asymptomatic influenza A virus (45 ng/mL, *p* = 0.19) and RSV (12 ng/mL, *p* = 0.05).

### ddhC concentration follows a similar time course to solicited symptoms and viral load in SARS-CoV-2 and influenza A virus infections

**Fig. 3:**
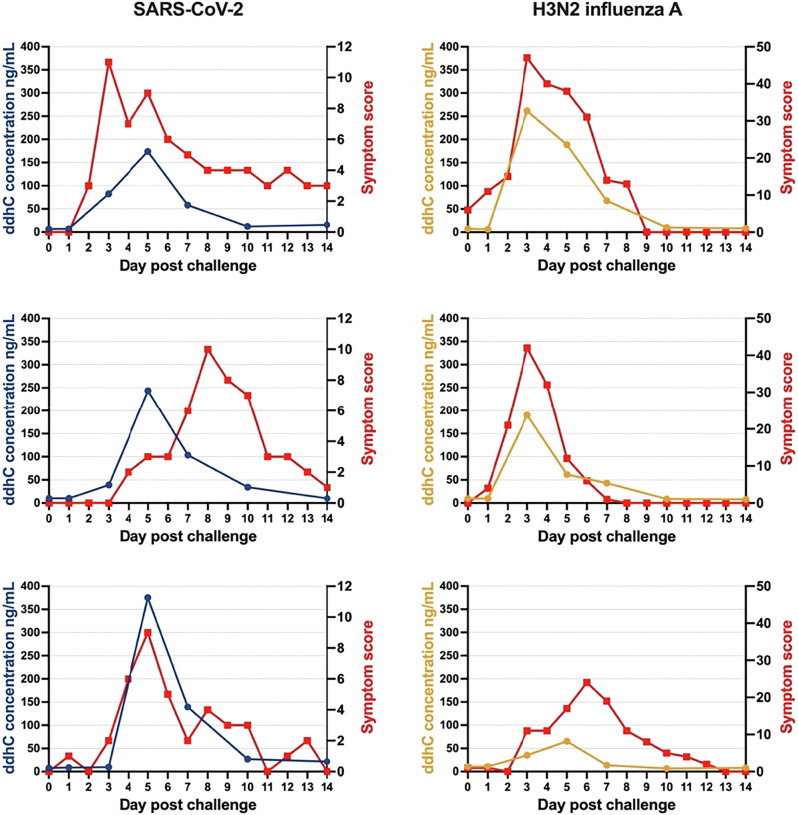
ddhC follows a similar time course to symptoms in symptomatic SARS-CoV-2 and influenza A virus challenge. Graphs show ddhC concentration (blue/yellow) and symptom data (red) from three individual symptomatic participants challenged with SARS-CoV-2 and three individual symptomatic participants challenged with influenza A virus. Symptom scores were generated using bespoke scoring scales in each study (Supplementary Table 1). Pearson’s correlation coefficients for matched timepoints are 0.58 (*p* < 0.01) for SARS-CoV-2 and 0.94 (*p* < 0.0001) for influenza A virus. While ddhC levels generally follow symptom trends, inter-individual differences exist.

In symptomatic participants infected with SARS-CoV-2 or influenza A virus, ddhC kinetics approximately aligned with the rise and fall in reported symptoms (Fig. 3; see Supplementary Fig. 3 for RSV symptom data), with Pearson’s correlation coefficients for matched timepoints of 0.58 (*p* < 0.01) for SARS-CoV-2 and 0.94 (*p* < 0.0001) for influenza A virus. In one participant infected with SARS-CoV-2 (middle panel, Fig. 3), the ddhC peak preceded the peak of reported symptoms. Different scales were used to score symptoms: for SARS-CoV-2, a bespoke symptom scale was used assessing 19 symptoms scored on a severity scale of 0–3 (evening score alone used in this study)^5^. For influenza A virus, a scale based on the Jackson symptom scoring system was used, assessing eight symptoms scored on a severity scale of 0–3 (combined am and pm score used in this study)^6,12^. No participants in either challenge study developed severe disease.

**Fig. 4:**
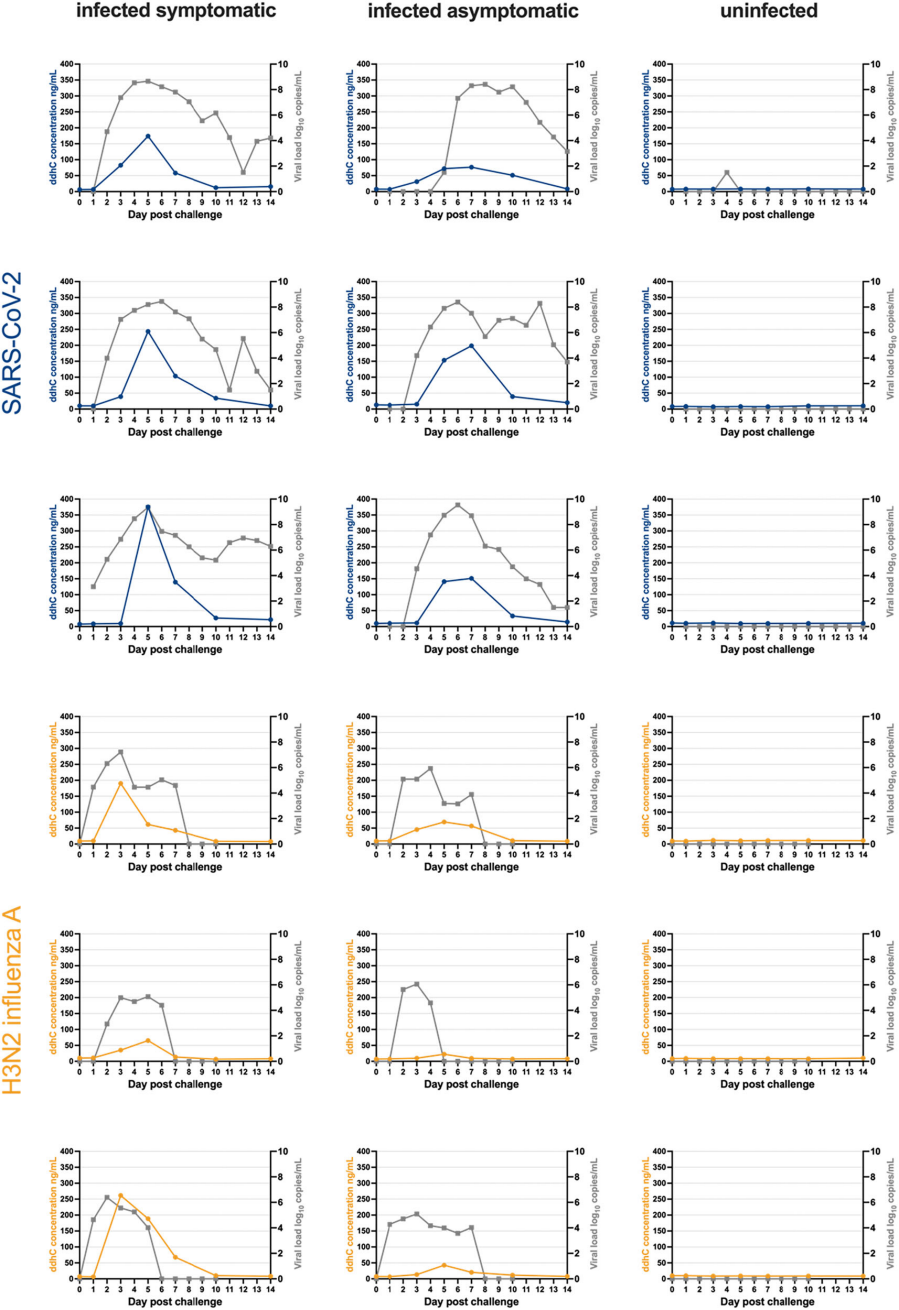
ddhC follows a similar time course to nasal viral load in SARS-CoV-2 and influenza A virus challenge. Each graph corresponds to an individual participant and shows ddhC concentration (blue/yellow) and nasal viral load (grey) over time. The left column represents symptomatic participants, the middle column asymptomatic/paucisymptomatic participants, and the right column uninfected participants. Nasal viral load was measured using nasal lavage in influenza A virus and RSV challenge, and using nasal swabs in SARS-CoV-2 challenge. Pearson’s correlation coefficients for matched timepoints are 0.69 (*p*-value < 0.0001) for SARS-CoV-2 and 0.57 (*p*-value < 0.0001) for influenza A virus.

ddhC concentrations displayed similar kinetics to nasal viral load in SARS-CoV-2 and influenza A virus challenge (Fig. 4; see Supplementary Fig. 4 for RSV nasal viral load data), with Pearson’s correlation coefficients for matched timepoints of 0.69 (*p*-value < 0.0001) and 0.57 (*p*-value < 0.0001), respectively. The area under the curves (AUCs) for ddhC concentration and nasal viral load for both SARS-CoV-2 and influenza A virus challenge also showed evidence of correlation, with Pearson’s correlation coefficients of 0.96 (*p*-value < 0.0001) and 0.70 (*p*-value < 0.05). Although influenza A virus and RSV studies used comparable methods of quantifying nasal viral load (through nasal lavage), the SARS-CoV-2 challenge study used nasal swabs and not nasal lavage, making direct comparison between challenge studies difficult.

### ddhC is associated with the interferon response

For SARS-CoV-2 and influenza A virus challenge studies, blood RNA-sequencing data from seven days were available for comparison with day-matched ddhC concentrations (days 0, 1, 3, 5, 7, 10 and 14 for SARS-CoV-2 and days 0, 1, 3, 7, 10, 14 and 28 for influenza A virus). For each participant timepoint (seven matching timepoints for each of the nine participants per study), normalised gene counts and ddhC concentrations were log_2_-transformed and correlation assessed using Pearson correlation coefficients.

**Fig. 5:**
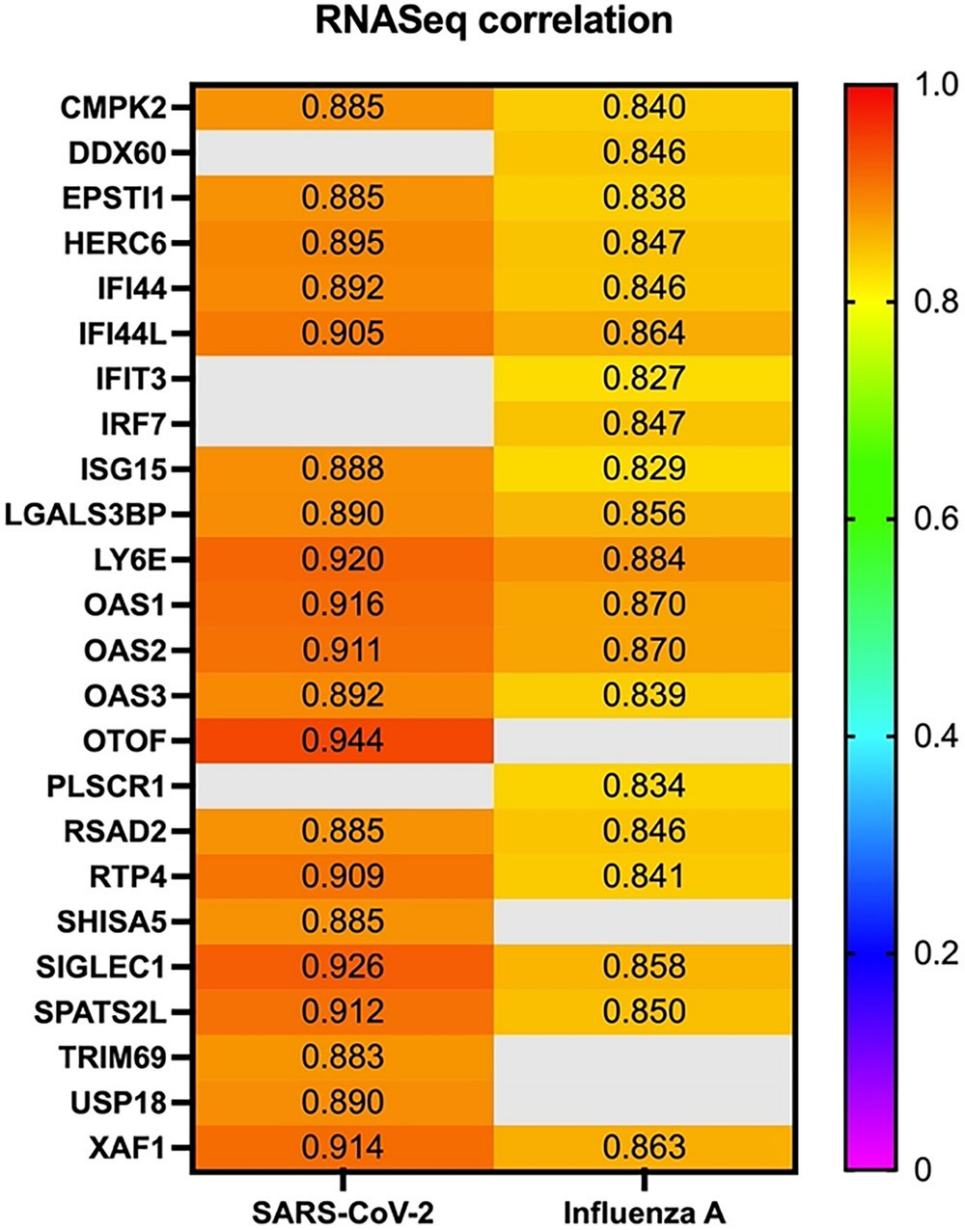
The 20 gene transcripts most highly correlated with ddhC in SARS-CoV-2 and influenza A virus challenge participants. Matched timepoints (where both plasma ddhC concentration and blood RNA sequencing data were available) from nine patients in each study were investigated. Pearson’s correlation between log_2_-transformed blood gene expression and log_2_-transformed plasma ddhC concentration was assessed, with the 20 most highly correlated genes shown.

Of >29,000 (SARS-CoV-2 challenge) and >18,000 (influenza A virus challenge) host gene transcripts measured, the top 20 transcripts that were most highly correlated with matched-day ddhC concentration were identified. 16 of the 20 genes were the same in both SARS-CoV-2 and influenza A virus challenged participants (Fig. 5). All 20 genes for both viruses are implicated in the interferon response, either as interferon-stimulated genes (ISGs) or involved in interferon regulation (Supplementary Table 3).

RSAD2, also known as viperin, and the adjacent gene CMPK2, which are both directly implicated in ddhCTP production^1^, were amongst the top 20 most correlated genes in both infections, with correlation coefficients ranging from 0.840 to 0.885 (*p*-values < 0.0001, Supplementary Fig. 5). The expression of viperin and CMPK2 over time followed a similar pattern to ddhC concentration (Supplementary Fig. 6).

## Discussion

Understanding the kinetics of a biomarker is a vital aspect of assessing its utility. A biomarker that is measurable for only a few minutes or hours has a different use case to one that remains elevated for several weeks. Generating data on a biomarker over time during natural infection is difficult, as samples are rarely available from both the time of exposure and at multiple time points over the course of infection. Until the current study, kinetic data on the recently described pan-viral biomarker 3’-deoxy-3′,4′-didehydro-cytidine (ddhC)^2–4^ have been lacking.

In this study, we capitalised on the strengths of human challenge studies, where close monitoring of infection is possible from the time of inoculation, including pre-inoculation timepoints. We showed that in non-severe infection following SARS-CoV-2 and H3N2 influenza A viral challenge, ddhC concentration follows an acute phase pattern over time, rising in the first few days of infection and falling to baseline between days 10 and 14 post inoculation.

A host-derived viral biomarker that peaks in the first few days of infection and subsequently returns to baseline is of great use in acute healthcare settings as an indicator of an active acute viral infection. Currently, in the absence of widespread rapid diagnostic capability, many patients presenting with non-specific symptoms of infection (e.g., fever, cough) are given antibiotics, even when they have a viral infection, driving antibiotic resistance^13^. Clinicians can use a rapid viral biomarker to identify patients with acute viral infection, which would aid in reducing the prescription of unnecessary antibiotics. Secondly, during the early stages of a novel viral pandemic, before pathogen-specific diagnostics were available, an acute pan-viral biomarker would be invaluable in helping diagnose and isolate patients with viral infections. The lack of early rapid viral diagnostic capabilities was a key factor in the exponential spread of COVID-19^14,15^. Thirdly, a sensitive and specific pan-viral host-derived biomarker such as ddhC would also provide a useful ‘rule-out’ diagnostic that could exclude active viral infection. This would be helpful when using highly sensitive molecular tests that can detect the presence of viral RNA or DNA and potentially mislead clinicians into assuming that a patient’s illness is related to active viral infection.

In this study, we investigated the ddhC response both in symptomatic and asymptomatic/paucisymptomatic infected participants. We showed, for the first time, that asymptomatic/paucisymptomatic infection does result in a ddhC response in SARS-CoV-2 and influenza A virus challenge, albeit not as high in concentration as in symptomatic infection. This capability to detect asymptomatic infection in a pandemic setting would be extremely useful. However, although in asymptomatic/paucisymptomatic SARS-CoV-2 infected participants the mean ddhC concentration was 142 ng/ml, it was 45 ng/ml in H3N2 influenza A virus infected participants, which was not significantly different than the mean of 30 ng/ml seen in symptomatic infected typhoid challenge controls. While this may limit ddhC’s utility as a differentiator in asymptomatic infection, whether this truly represents values seen in natural infection, or relates to infection dose and severity, is unknown, and direct comparison between the challenge studies is difficult given their different conditions.

It is unclear why a ddhC response was not seen in RSV challenge participants in this study, both in the younger and older participants. In previous work, we observed elevated ddhC levels in a patient hospitalised with RSV infection^2^. The RSV infections in this challenge study were notably mild^7^, and so it is possible that a more severe RSV infection is required to produce a detectable ddhC response. In the discovery patient cohort where ddhC was first identified as a viral biomarker, its levels correlated with viral disease severity (based on hospital admission duration, intensive care admission and mortality), though in a separate smaller cohort, it was not possible to fully validate this^2^. RSV is less cytopathic and pathogenic than SARS-CoV-2 and influenza A virus, known to modulate the immune response by suppressing type I interferon production whilst inducing interferon-λ production^16^, factors that may in turn affect the ddhC response. Additionally, potential differences in nasal viral replication between RSV challenge and natural infection could impact interferon signalling and the associated ddhC response. Further evaluation at different timepoints in patients with natural RSV infection, as well as in a broader range of viral infection severity, is required.

In this study, we were also able to interrogate whole blood RNA sequencing data and correlate gene counts with ddhC concentrations. We found that of the 20 genes whose counts were most highly correlated to ddhC concentrations, all are implicated in the interferon response (Supplementary Table 3). Furthermore, both RSAD2 (also known as viperin) and CMPK2, two genes implicated in ddhCTP production^1^, were amongst these top 20 most correlated genes, supporting this proposed metabolic pathway as the source of circulating ddhC in humans. Although transcriptomic signatures are being widely used to classify infection categories, a single small-molecule biomarker like ddhC offers distinct advantages. ddhC is detectable in easily accessible biological fluids such as serum, plasma and urine^3^. ddhC exhibits high stability^2^, and eliminates the need for gene extraction or amplification, making it a potentially more practical and efficient option for clinical applications. Efforts are underway to develop rapid, cost-effective, and easily deployable methods for detecting ddhC at the point-of-care, enabling broader access to this diagnostic tool.

This study should be viewed in the context of its limitations. First, the human challenge model, despite its strengths in controlled infection and monitoring, does not recapitulate all aspects of natural infection. Factors such as viral inoculum dose and mode of acquisition, as well as host factors, will affect the host immune response. Second, this study includes mainly younger adults (age range 20–52); whether ddhC kinetics are similar in older adults and children requires further investigation. Third, direct comparisons between the ddhC response in the three infections investigated are difficult, due to the different challenge conditions and methodologies in each study. Fourth, participant numbers included in this study are low, limited by access to LC-MS/MS assays and sample availability. Similarly, since ddhC was measured on alternate days, the data lack the granularity required for more precise comparisons with daily symptom and viral load data. Fifth, the majority of participants were of white ethnicity; more data in patients from other ethnicities are needed to identify any potential effect of host genetic variability on this biomarker. Future studies evaluating ddhC kinetics will include more diverse populations (varying in ethnicity, age, and comorbidities), different healthcare settings (primary versus secondary care), and a comparison of natural infection with human challenge models.

In conclusion, using human challenge infection models, we showed for the first time that the viral biomarker ddhC acts as an acute-phase reactant in SARS-CoV-2 and H3N2 influenza A virus infection. This pattern can be seen in both symptomatic and asymptomatic/paucisymptomatic infected individuals. These data add support to the potential use of ddhC as a biomarker for acute viral infection and serve as a salutary reminder that sample timing is critical when researching infection biomarkers.

## Supplementary information

Supplementary Information

## Data Availability

Data are provided within the manuscript or supplementary information files.
