# Supplementary material for: Longitudinal kinetics of the viral infection biomarker 3′-deoxy-3′,4′-didehydro-cytidine in SARS-CoV-2, influenza A virus and RSV human challenge models
Source: Npj Viruses. 2025 Jun 20;3:50. doi: 10.1038/s44298-025-00132-x (PMC12181374; doi:10.1038/s44298-025-00132-x)
Supplement: Supplementary file 1 — Supplementary Information [file 44298_2025_132_MOESM1_ESM.docx]

**Longitudinal kinetics of the viral infection biomarker 3’-deoxy-3’,4’-didehydro-cytidine in SARS-CoV-2, influenza A virus and RSV human challenge models**

**Supplementary Information**

[Table 1. Symptom scores for each participant. 2](#_Toc194502760)

[Table 2. Participant demographics. 3](#_Toc194502761)

[Table 3. Genes correlated to ddhC and their association with the interferon response pathway in SARS-CoV-2 and influenza A challenge participants. 4](#_Toc194502762)

[Figure 1. ddhC concentrations in three participants from the older arm of the RSV challenge study. 5](#_Toc194502763)

[Figure 2. The ddhC response to human challenge with Salmonella Typhi in 10 participants. 6](#_Toc194502764)

[Figure 3. Symptoms compared with ddhC concentration in symptomatic RSV challenge participants. 7](#_Toc194502765)

[Figure 4. Nasal viral load in comparison to ddhC concentration in RSV challenge participants. 8](#_Toc194502766)

[Figure 5. Correlation between ddhC concentration and viperin / CMPK2 gene expression in SARS-CoV-2 and influenza A challenge participants. 9](#_Toc194502767)

[Figure 6. The expression of viperin and CMPK2 follow a similar pattern to ddhC concentration over time. 10](#_Toc194502768)

[ddhC targeted LC-MS/MS assay 11](#_Toc194502769)

[Chemicals and materials 11](#_Toc194502770)

[LC-MS setup 11](#_Toc194502771)

[Serum and plasma samples for method validation 11](#_Toc194502772)

[Surrogate and zero matrix 12](#_Toc194502773)

[Figure 7. Total ion current chromatograms (TIC) obtained from global HILIC profiling in positive ionisation mode. 12](#_Toc194502774)

[Preparation of the calibration and quality control working solutions 13](#_Toc194502775)

[Calibration solutions and QC samples preparation for LC-MS/MS analysis 13](#_Toc194502776)

[Samples formatting and run order 14](#_Toc194502777)

[MS/MS transitions 15](#_Toc194502778)

[Table 4. MS/MS transitions used for ddhC and its labelled ^13^C_5_-ddhC standard. 15](#_Toc194502779)

[Method validation 15](#_Toc194502780)

[Table 5. Comparison of the calibration curve generated in surrogate matrix to the standard addition calibration curve in native matrix (LTR-P) and its dilution series. 16](#_Toc194502781)

[Table 6. Comparison of the calibration curve generated in zero matrix to the standard addition calibration curve in native matrix (LTR-P) and its dilution series. 17](#_Toc194502782)

[Table 7. Intra- (n=3) and Inter-day (n=13) % accuracy and precision as (%CV) for five QC concentrations and precision for LTR-P and LTR-S. 18](#_Toc194502783)

[Table 8. ME assessment results. 20](#_Toc194502784)

[Table 9. Stability assessment results. 21](#_Toc194502785)

[Table 10. Dilution integrity assessment results. 22](#_Toc194502786)

[Data processing 22](#_Toc194502787)

[References 23](#_Toc194502788)

| Challenge virus | Participant category | Symptom score |
| --- | --- | --- |
| SARS-CoV-2 | infected symptomatic | 73 |
| SARS-CoV-2 | infected symptomatic | 48 |
| SARS-CoV-2 | infected symptomatic | 40 |
| SARS-CoV-2 | infected asymptomatic/paucisymptomatic | 3 |
| SARS-CoV-2 | infected asymptomatic/paucisymptomatic | 1 |
| SARS-CoV-2 | infected asymptomatic/paucisymptomatic | 0 |
| SARS-CoV-2 | uninfected | n/a |
| SARS-CoV-2 | uninfected | n/a |
| SARS-CoV-2 | uninfected | n/a |
| H3N2 influenza A | infected symptomatic | 244 |
| H3N2 influenza A | infected symptomatic | 118 |
| H3N2 influenza A | infected symptomatic | 115 |
| H3N2 influenza A | infected asymptomatic/paucisymptomatic | 28 |
| H3N2 influenza A | infected asymptomatic/paucisymptomatic | 13 |
| H3N2 influenza A | infected asymptomatic/paucisymptomatic | 0 |
| H3N2 influenza A | uninfected | n/a |
| H3N2 influenza A | uninfected | n/a |
| H3N2 influenza A | uninfected | n/a |
| RSV | infected symptomatic | yes |
| RSV | infected symptomatic | yes |
| RSV | infected asymptomatic/paucisymptomatic | no |
| RSV | uninfected | n/a |
| RSV | uninfected | n/a |
| RSV | uninfected | n/a |

### Table 1. Symptom scores for each participant.

Symptom score scales varied between studies - for SARS-CoV-2, a bespoke symptom scale was used assessing 19 symptoms scored on a severity scale of 0-3 (evening score alone used in this study);^1^ for influenza A, a scale based on the Jackson symptom scoring system was used, assessing eight symptoms scored on a severity scale of 0-3 (combined am and pm score used in this study);^2,3^ for RSV, an adapted Jackson scoring system was used as follows to define whether participants were symptomatic or not: “Participants were defined as symptomatic if they fulfilled two out of three criteria: a cumulative 14-day symptom score of 14 or more, subjective feeling of cold, or nasal discharge for at least 3 days.”

|  | Total participants included (n) | Age  (median [range]) | Female (n) | Ethnicity | | | |
| --- | --- | --- | --- | --- | --- | --- | --- |
|  |  |  |  | **White** | **Asian** | **Chinese** | **Mixed** |
| SARS-CoV-2 | 9 | 21 [20-27] | 4 | 8 | 0 | 0 | 1 |
| H3N2 influenza A | 9 | 44.5 [26-53] | 4 | 7 | 1 | 1 | 0 |
| RSV | 6 | 34.5 [24-52] | 1 | 4 | 1 | 0 | 1 |
| Typhoid | 10 | 27.4 [24-32] | 4 | 9 | 0 | 0 | 1 |

### Table 2. Participant demographics.

| Gene | SARS-CoV-2 correlation | Influenza A correlation | Association with interferon pathway | Source |
| --- | --- | --- | --- | --- |
| CMPK2 | 0.885 | 0.84 | ISG | GeneCards |
| DDX60 |  | 0.846 | ISG | GeneCards |
| EPSTI1 | 0.885 | 0.838 | ISG | Buang et al., 2021 (Nature Communications)^4^ |
| HERC6 | 0.895 | 0.847 | ISG | Uppala et al., 2024 (iScience)^5^ |
| IFI44 | 0.892 | 0.846 | ISG | Busse et al., 2020 (J Virol)^6^ |
| IFI44L | 0.905 | 0.864 | ISG | Busse et al., 2020 (J Virol)^6^ |
| IFIT3 |  | 0.827 | ISG | GeneCards |
| IRF7 |  | 0.847 | IFN transcriptional regulator | GeneCards |
| ISG15 | 0.888 | 0.829 | ISG | GeneCards |
| LGALS3BP | 0.89 | 0.856 | ISG | De Jarcy et al., 2023 (Clin Exp Med)^7^ |
| LY6E | 0.92 | 0.884 | ISG | Mar et al. 2018 (Nature Communications)^8^ |
| OAS1 | 0.916 | 0.87 | ISG | GeneCards |
| OAS2 | 0.911 | 0.87 | ISG | GeneCards |
| OAS3 | 0.892 | 0.839 | ISG | GeneCards |
| OTOF | 0.944 |  | ISG | Ding et al. 2022 (mBio)^9^ |
| PLSCR1 |  | 0.834 | ISG | GeneCards |
| RSAD2 | 0.885 | 0.846 | ISG | GeneCards |
| RTP4 | 0.909 | 0.841 | IFN transcriptional regulator | He et al. 2020 (PNAS)^10^ |
| SHISA5 (SCOTIN) | 0.885 |  | ISG | Kim et al. 216 (Nature Communications)^11^ |
| SIGLEC1 | 0.926 | 0.858 | ISG | Zheng et al. 2015 (Cell Res)^12^ |
| SPATS2L | 0.912 | 0.85 | IFN transcriptional regulator | Chen et al. 2024 (ABBS)^13^ |
| TRIM69 | 0.883 |  | ISG | Wang et al. 2018 (PLOS Path)^14^ |
| USP18 | 0.89 |  | ISG | GeneCards |
| XAF1 | 0.914 | 0.863 | ISG | Jeong et al. 2018 (Cell Death Dis)^15^ |

###

### Table 3. Genes correlated to ddhC and their association with the interferon response pathway in SARS-CoV-2 and influenza A challenge participants.

The 20 genes most highly correlated with ddhC concentration in both SARS-CoV-2 and H3N2 influenza A challenge are all implicated in the interferon response pathway. Pearson correlation coefficients are shown. ISG = interferon stimulated gene. IFN = interferon. GeneCards refers to the Human Gene database (genecards.org).

### Figure 1. ddhC concentrations in three participants from the older arm of the RSV challenge study.

Results are shown as mean ± s.e.m. for the three participants.


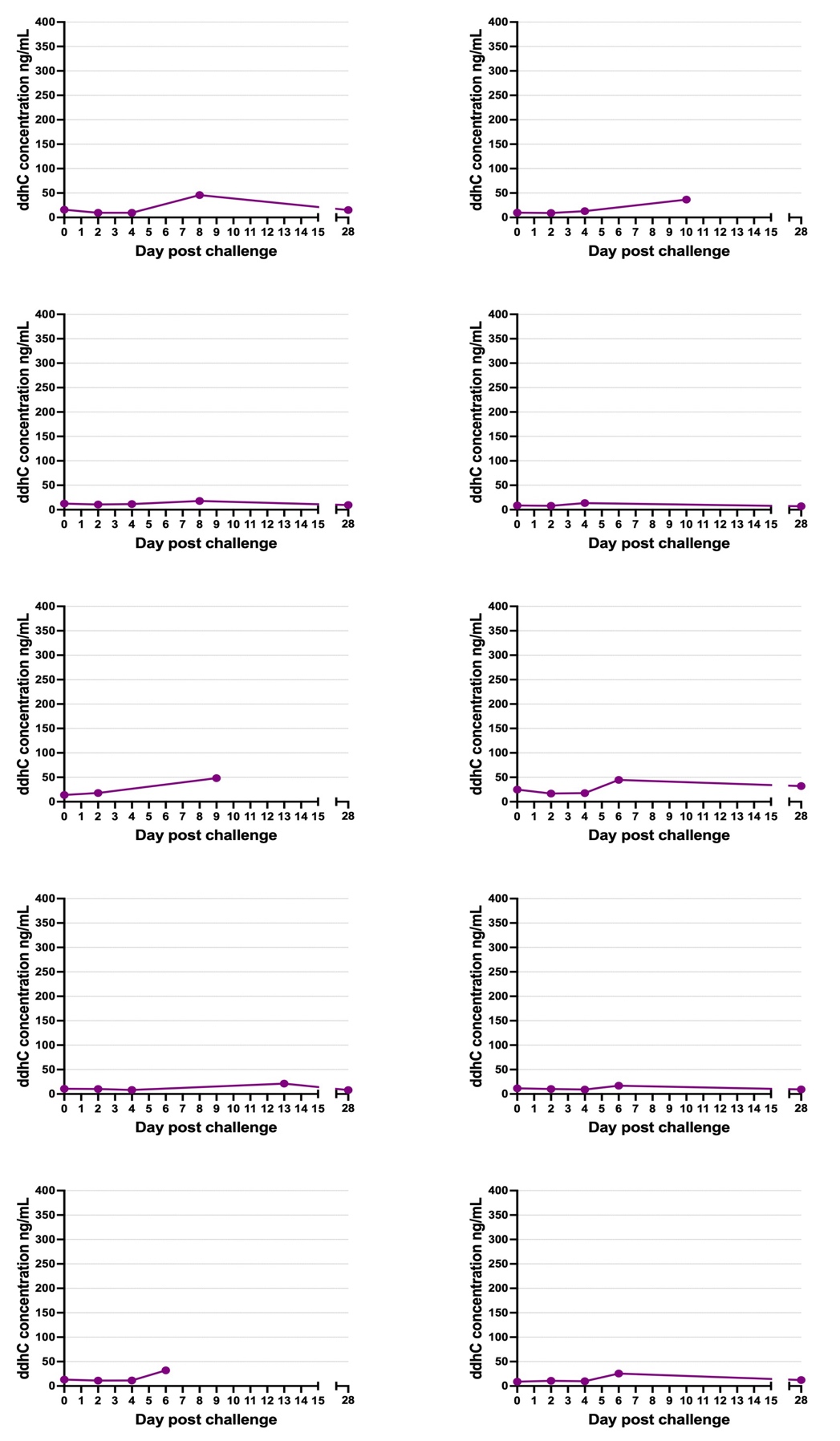


### Figure 2. The ddhC response to human challenge with Salmonella Typhi in 10 participants.

The maximum ddhC concentration in each patient coincided with the day of typhoid diagnosis (fever and/or bacteraemia). Several timepoint samples were unavailable for testing. “D0” refers to 12 hours post challenge.


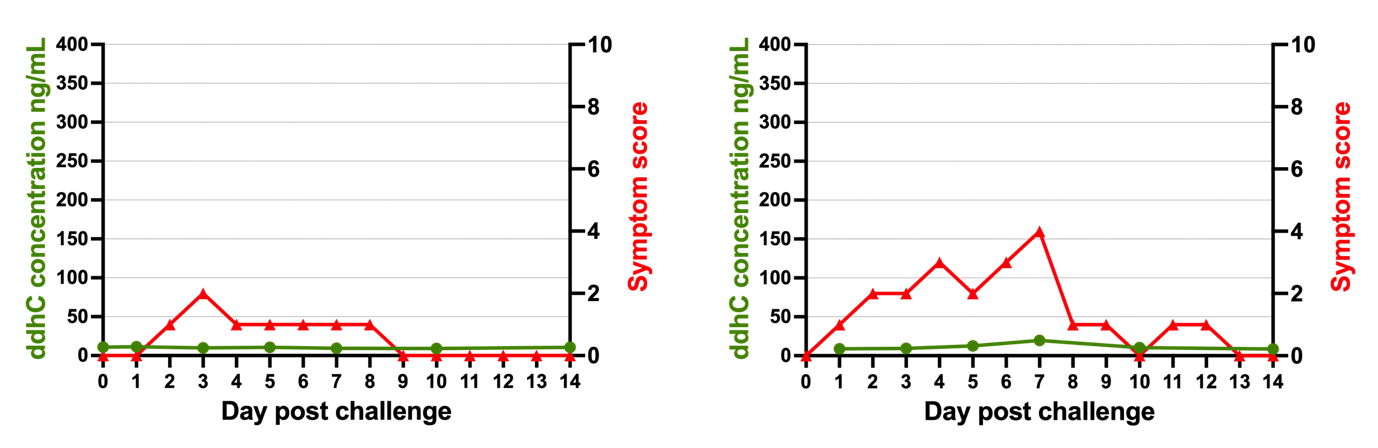


### Figure 3. Symptoms compared with ddhC concentration in symptomatic RSV challenge participants.

Each graph corresponds to an individual participant and shows ddhC concentration (green) and reported symptoms (red) over time. Symptoms were scored using an adapted Jackson scoring system based on eight symptoms graded 0-3.


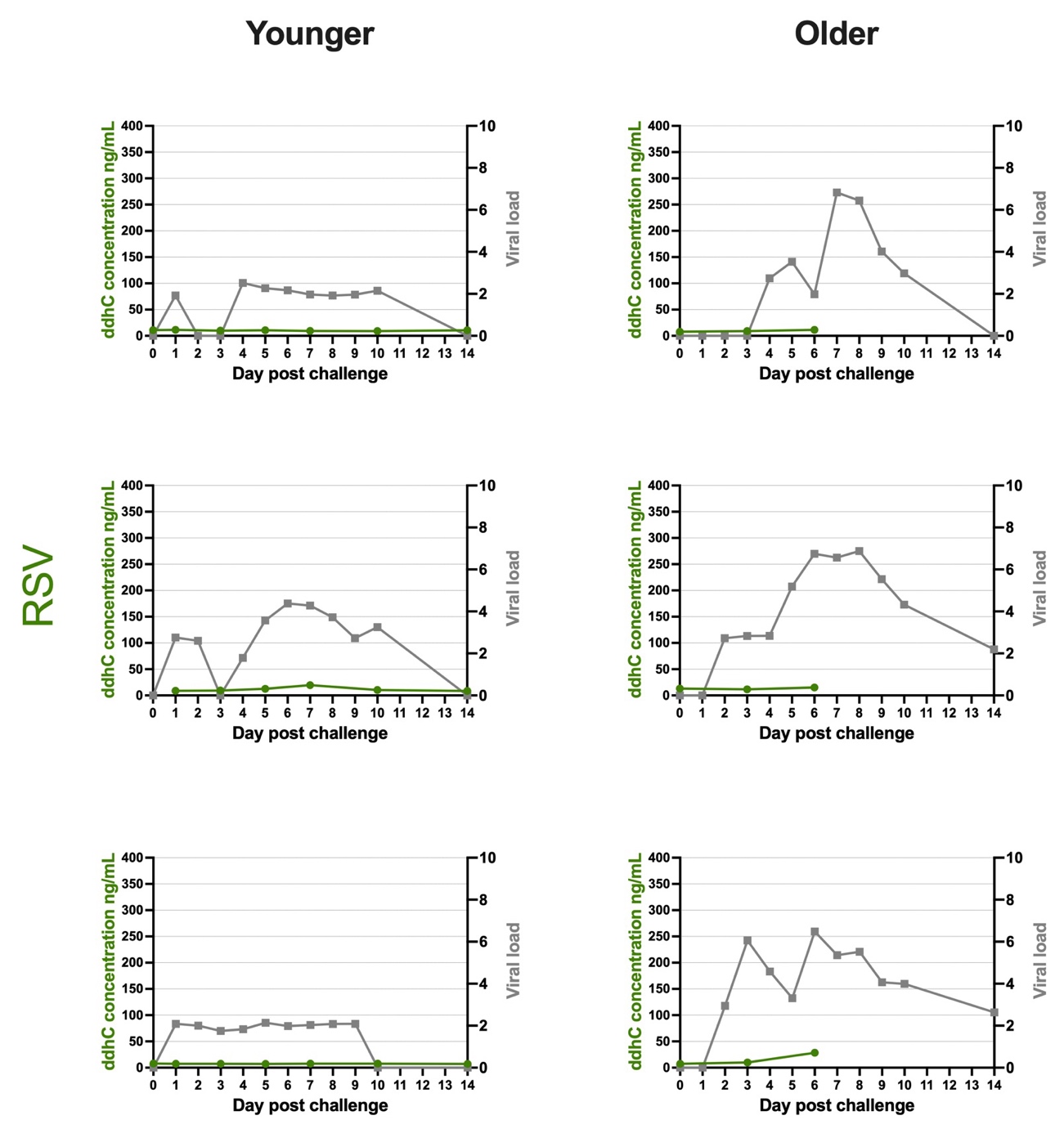


### Figure 4. Nasal viral load in comparison to ddhC concentration in RSV challenge participants.

Each graph corresponds to an individual participant and shows ddhC concentration (green) and nasal viral load (grey) over time. The left and right columns represent infected participants from the younger (aged 18-55 years) and older (aged 60-75 years) arms of the RSV challenge study, respectively. Nasal viral load was measured using nasal lavage.


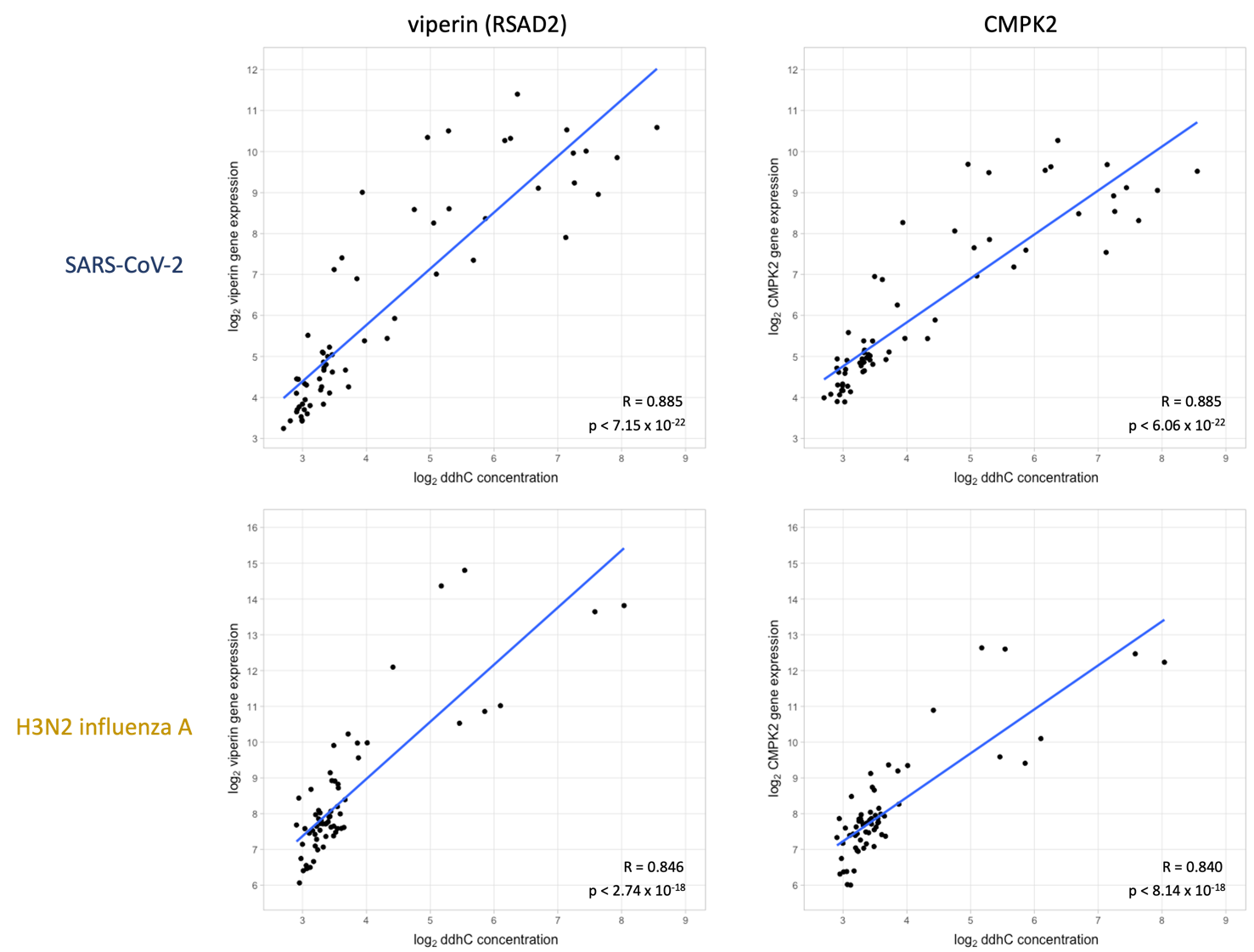


### Figure 5. Correlation between ddhC concentration and viperin / CMPK2 gene expression in SARS-CoV-2 and influenza A challenge participants.

Each dot represents a specific timepoint sample for an individual participant; seven timepoints for each of the nine participants from both studies were assessed (n = 63 matched timepoints per study). Pearson correlation coefficients and corresponding p-values are shown.


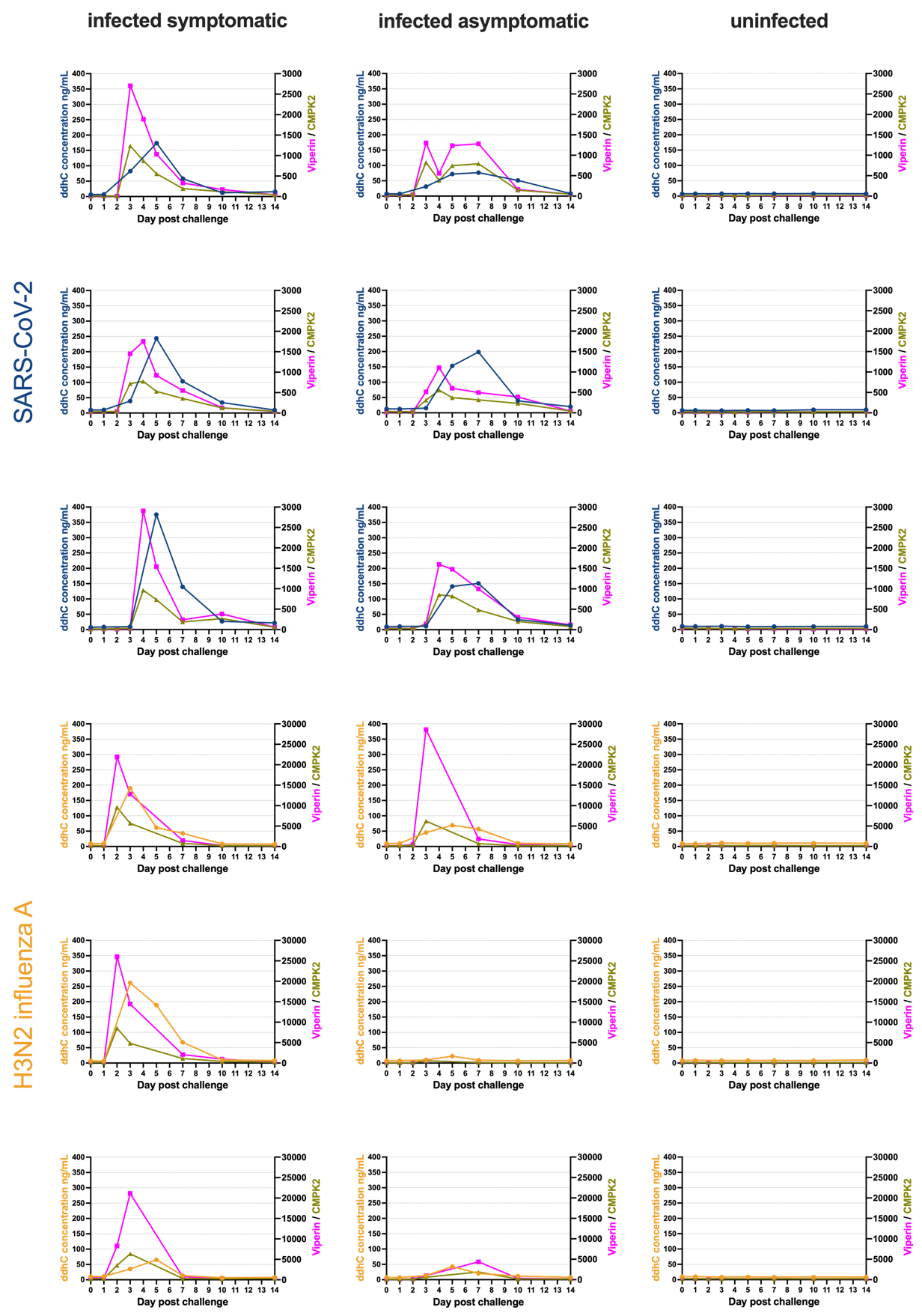


### Figure 6. The expression of viperin and CMPK2 follow a similar pattern to ddhC concentration over time.

Data are shown for all SARS-CoV-2 and influenza A challenge participants. Graphs show ddhC concentration (blue/yellow) and viperin (pink) and CMPK2 expression (green). The left column represents symptomatic participants, the middle column asymptomatic/paucisymptomatic participants, and the right column uninfected participants.

## ddhC targeted LC-MS/MS assay

### Chemicals and materials

LC-MS grade acetonitrile with formic acid (0.1%, v/v), LC-MS grade water with formic acid (0.1%, v/v), and LC-MS grade water were purchased from Fisher Scientific (Loughborough, U.K.), LC-MS grade acetonitrile was purchased from VWR International Ltd (Leicestershire, UK). Ammonium formate, CHROMASOLV LC-MS Ultra was obtained from SLS Scientific Laboratory Supplies Limited (Fairham, UK). Oasis® HLB Vac Cartridge (30 mg, 1cc) for zero matrix generation were from Waters (Wilmslow, UK). For generating the surrogate matrix, bovine serum albumin (BSA) and phosphate buffered saline (PBS, pH 7.4) were obtained from Sigma-Aldrich (Gillingham, UK).

### LC-MS setup

### Serum and plasma samples for method validation

For the assay validation various serum and plasma samples were used. Pooled long-term reference (LTR) human serum and plasma samples, LTR-S and LTR-P respectively, maintained by the National Phenome Centre (NPC) and used as independent sample references^16^ were purchased from Seralab (BioIVT) as bulk human serum and plasma (10L of each that were homogenised, centrifuged and aliquoted for long-term storage).

In addition to the LTR samples, plasma samples were collected from six healthy controls [ethics approval reference 12/WA/0196] and used for assessing selectivity and matrix effect after removing ddhC from the samples using the SPE approach described in the next section.

To compare ddhC levels in two different blood products, serum and plasma, same-day paired samples were retrieved from the Northwest London Pathology clinical laboratory from 11 patients with PCR- or antigen-confirmed viral infection (ethics approval reference 06/Q0406/20).

### Surrogate and zero matrix

Surrogate matrix was prepared by weighing BSA and dissolving it in PBS (pH 7.4) to prepare a solution of 2% [w/v]. Surrogate matrix was used in all analyses for the preparation of calibration and quality control (QC) solutions.

Zero matrix – a sample matrix lacking the analyte of interest or analyte-free matrix – was used to assess matrix effect and selectivity. It was prepared by solid phase extraction (SPE) using an Oasis® HLB Vac Cartridge (30 mg, 1cc) conditioned with LC-MS grade acetonitrile (1 mL) and then equilibrated with LC-MS water (1 mL). After the conditioning and equilibration, an aliquot of 300 μL of plasma samples (LTR-P and healthy control plasma) diluted 1:1 with LC-MS grade water was loaded onto the cartridge. The loading solution was collected in an Eppendorf tube. The cartridge was washed twice with LC-MS grade water (300 μL) collecting the wash solution in a separate Eppendorf tube. Following this step, the elution of the retained material was done sequentially using 1:1 acetonitrile:water twice (300 μL) and then acetonitrile twice (300 μL). The loading solution lacked ddhC but retained the global plasma/serum metabolic profile as the untreated sample when analysed by hydrophilic interaction liquid chromatography (HILIC) assay used at the NPC^16^ as shown in (Figure 7). The SPE process was repeated using the same cartridge by passing through the collected loading solution. The new loading solution (after the second SPE) was collected into a new Eppendorf tube and was used in the further validation analyses as a zero matrix (with removed endogenous ddhC).

Figure 7. Total ion current chromatograms (TIC) obtained from global HILIC profiling in positive ionisation mode. **A.** TIC of the LTR serum load fraction collected from the HLB oasis cartridge. **B.** TIC of the test LTR serum before SPE. The X-axes represent time (min), ranging from 0.4 min to 7.2 min. The Y-axis represent the relative intensity of ion signals, ranging from 0% to 100%. Numerical labels represent peak elution time (top) and m/z ratio (bottom).

### Preparation of the calibration and quality control working solutions

Stock solution of ddhC of 1 mg/mL was prepared in LC-MS grade water and stored at -80°C in 200 µL aliquots. SIL-IS ^13^C_5_-ddhC stock was prepared at 1 mg/mL in LC-MS grade water, then it was subaliquoted at a concentration of 600 ng/mL. The multiple aliquots of 600 ng/mL were stored at -80°C, and the needed number of aliquots were thawed prior to each analysis.

A set of ten calibration working solutions (WS) of ddhC in the defined validated linear range (3.9-2000 ng/mL) was prepared from the stock solution of ddhC by serial dilution with LC-MS grade water using a dilution factor of two. WS of the quality control (QC) samples were prepared separately, diluting the ddhC stock solution with LC-MS grade water to five concentration levels of ddhC, that were within the linear range but different from any calibration WS concentration levels, except for the lower limit of quantification (LLOQ): 3.9 ng/mL (LLOQ QC), 50 ng/mL, 300 ng/mL, 625 ng/mL, and 1200 ng/mL (high QC).

### Calibration solutions and QC samples preparation for LC-MS/MS analysis

Preparation of the calibration solutions and QC samples for the LC-MS/MS analysis was done using the same procedure as for the preparation of serum and plasma samples starting with aliquots of 40 μL of each WS in LC-MS water, which were diluted 1:1 with surrogate matrix of 2% BSA in PBS. This was followed by the addition of 16 μL of 600 ng/mL aqueous solution of ^13^C_5_-ddhC SIL-IS. Three volumes of ice-cold acetonitrile (288 μL) were added to 96 μL of calibration WS or QC solution mixed with the surrogate matrix and SIL-IS for protein precipitation. After the mixing at 1400rpm for 2h at 4°C and centrifugation for 10 minutes at 3486×g and 4°C, the supernatant was collected for the targeted LC-MS/MS analysis.

Double blank (DB) solution was prepared as method diluent containing 1:3 LC-MS grade water:acetonitrile. Single blank (SB) was prepared as described above for calibration solutions but using water instead of the calibration WS diluting 1:1 with the surrogate matrix and adding the same amount of SIL-IS and ice-cold acetonitrile. SB sample was mixed and centrifuged as described for the calibration and QC solutions. It was analysed as “zero point” calibration solution before the LLOQ solution but not included in the calibration linear fit regression.

### Samples formatting and run order

Clinical samples were analysed as randomised blocks of samples collected from each patient in different days of infection. Additionally, the established run order randomized the samples from different days of infection within those sample blocks to avoid any confounding effects of clinical variables to the analytical run order.

The samples were extracted as described above and aliquoted onto two analytical plates. The first analytical plate comprised 40 study samples and the second 33 study sample. Each plate included a set of ten calibration solutions with SB solution preceding it, and two full set of QC samples. LTR-P samples were added to the run to monitor the precision of ddhC quantification in a repeat analysis of a biological sample. DB solutions were aliquoted in the column 12 of the analytical plate.

The analytical was initiated by the triple injection of the DB solution to equilibrate the column followed by a SB injection, calibration set and one full QC series. The study samples from two analytical plates were analysed continuously in subsets of eight samples. LTR-P sample was injected before each subset to monitor the precision of ddhC quantification across the full run. The calibration and analytical QC set were repeated at the end of the analysis of all study samples. DB was injected in duplicate after each ULOQ solution and high QC to avoid any carryover in the subsequent samples.

For method validation, performed on different days, each analytical plate contained the required number of calibration and QC sets according to the requirements for each validation parameter described in the next section.

### MS/MS transitions

| **Analyte** | **Parent *m/z*** | **Quantifier *m/z*** | **Qualifier *m/z*** | **Collision Energy, eV** |
| --- | --- | --- | --- | --- |
| ddhC | 225.97 | 112.04 |  | 16 |
|  | 225.97 |  | 190.12 | 10 |
|  | 225.97 |  | 147.08 | 16 |
|  | 225.97 |  | 95.00 | 34 |
|  | 225.97 |  | 81.02 | 24 |
| ddhC-^13^C_5_ | 231.07 | 94.97 |  | 34 |
|  | 231.07 |  | 112.07 | 18 |

### Table 4. MS/MS transitions used for ddhC and its labelled ^13^C_5_-ddhC standard.

### Method validation

The method validation was based on the established guidelines for bioanalytical method validation.^17,18^

Parallelism study

Prior to the full method validation, we conducted a parallelism study to demonstrate the suitability of the surrogate matrix (2% (w/v) of BSA in PBS (pH 7.4)) to perform as native matrix in the full method validation and all future applications for the analysis of ddhC in human serum and plasma samples.^19^ The calibration sets were prepared in triplicate using LTR-P for the standard addition method and surrogate matrix for an external calibration to dilute 1:1 the calibration WS as described above. In addition, a dilution series was prepared in triplicate serially diluting LTR-P by the factor of 1.5 three times. The concentration of ddhC in LTR-P was obtained by extrapolating the standard addition calibration curves prepared in LTR-P. The concentrations of ddhC in the three diluted LTR-P samples were calculated subsequently from obtained extrapolated ddhC concentration in LTR-P. Finally, to assess the suitability of the surrogate matrix for an accurate and precise analysis of ddhC in biological matrix, each diluted and spiked with standard addition LTR-P solution were quantified against the external calibration curves constructed in surrogate matrix. These interpolated ddhC concentrations were compared to the ddhC concentration in LTR-P obtained by the standard addition method and the calculated concentrations of ddhC in the diluted and spiked solutions of LTR-P. The slopes of both sets of calibration curves were compared. The precision of all measured (extrapolated and interpolated) ddhC concentrations were calculated as %RSD in the spiked and diluted LTR-P solutions analysed in triplicate. The accuracy of the interpolated ddhC concentrations was calculated for each spiked and diluted concentration levels by comparing to the extrapolated and calculated ddhC levels (Table 5).

The parallelism study was repeated using zero-matrix, prepared using SPE as described above, to compare with the standard addition method and external calibration in surrogate matrix (Table 6). Having proven the parallel behaviour of the surrogate matrix to the native and zero matrix and its general accessibility and availability, which would allow method transfer into and between clinical and analytical labs, the method of surrogate matrix was chosen for further method validation and analyses of biological samples.

|  | **ddhC*** | **Interp-olated 1** | **Interp-olated 2** | **Interp-olated 3** | **%CV** | **%Dev1** | **%Dev2** | **%Dev3** | **Average %Dev** |
| --- | --- | --- | --- | --- | --- | --- | --- | --- | --- |
| DS4 | 2.3 | 2.0 | 2.2 | NA | 9.0% | -15.5% | -4.0% | NA | -9.8% |
| DS3 | 3.5 | 3.9 | 2.9 | 3.0 | 16.2% | 11.7% | -15.8% | -13.5% | -5.9% |
| DS2 | 5.2 | 5.1 | 4.9 | 5.1 | 2.1% | -1.9% | -5.8% | -2.5% | -3.4% |
| DS1 | 7.8 | 7.1 | 7.7 | 8.2 | 7.1% | -9.0% | -1.1% | 4.8% | -1.7% |
| Cal00 | 11.7 | 12.1 | 11.6 | 12.0 | 2.2% | 3.7% | -0.7% | 2.6% | 1.9% |
| Cal01 | 13.6 | 14.4 | 14.0 | 14.2 | 1.7% | 5.8% | 2.3% | 4.1% | 4.1% |
| Cal02 | 15.6 | 16.8 | 18.4 | 15.2 | 9.3% | 7.9% | 17.6% | -2.4% | 7.7% |
| Cal03 | 19.5 | 20.7 | 20.8 | 20.7 | 0.2% | 6.2% | 6.4% | 6.1% | 6.2% |
| Cal04 | 27.3 | 31.0 | 29.9 | 30.4 | 1.8% | 13.5% | 9.6% | 11.3% | 11.5% |
| Cal05 | 42.9 | 50.2 | 50.2 | 45.6 | 5.5% | 16.9% | 16.9% | 6.1% | 13.3% |
| Cal06 | 74.2 | 80.5 | 86.2 | 75.5 | 6.6% | 8.5% | 16.2% | 1.8% | 8.8% |
| Cal07 | 136.7 | 148.8 | 142.0 | 138.6 | 3.6% | 8.9% | 3.9% | 1.4% | 4.7% |
| Cal08 | 261.7 | 263.8 | 300.5 | 307.3 | 8.1% | 0.8% | 14.8% | 17.4% | 11.0% |
| Cal09 | 511.7 | 545.6 | 504.6 | 561.6 | 5.5% | 6.6% | -1.4% | 9.7% | 5.0% |
| Cal10 | 1011.7 | 998.8 | 1015.2 | 1022.4 | 1.2% | -1.3% | 0.3% | 1.1% | 0.0% |
| Cal11 | 2011.7 | 2051.3 | 2125.2 | 2065.0 | 1.9% | 2.0% | 5.6% | 2.7% | 3.4% |

* Extrapolated concentration of ddhC (LTR-P (from standard addition)

### Table 5. Comparison of the calibration curve generated in surrogate matrix to the standard addition calibration curve in native matrix (LTR-P) and its dilution series.

Comparison of the concentration values measured for each standard addition point in LTR plasma matrix and dilution series from the calibration curve generated in surrogate matrix to the concentration values extrapolated from the standard addition calibration curves and calculated concentration of diluted LTR plasma sample.

|  | **ddhC conc.*** | **Interp-olated 1** | **Interp-olated 2** | **Interp-olated 3** | **%CV** | **%Dev1** | **%Dev2** | **%Dev3** | **Average %Dev** |
| --- | --- | --- | --- | --- | --- | --- | --- | --- | --- |
| DS4 | 2.3 | 2.1 | 2.4 | NA | 7.8% | -8.5% | 2.2% | NA | -3.1% |
| DS3 | 3.5 | 3.9 | 3.0 | 3.1 | 14.7% | 12.4% | -13.1% | -11.0% | -3.9% |
| DS2 | 5.2 | 5.0 | 4.8 | 5.0 | 2.0% | -3.2% | -6.7% | -3.7% | -4.6% |
| DS1 | 7.8 | 6.9 | 7.5 | 7.9 | 6.8% | -11.7% | -4.4% | 1.1% | -5.0% |
| Cal00 | 11.7 | 11.5 | 11.1 | 11.4 | 2.2% | -1.3% | -5.3% | -2.2% | -2.9% |
| Cal01 | 13.6 | 13.7 | 13.2 | 13.5 | 1.7% | 0.4% | -3.0% | -1.3% | -1.3% |
| Cal02 | 15.6 | 15.9 | 17.3 | 14.4 | 9.1% | 2.0% | 11.0% | -7.6% | 1.8% |
| Cal03 | 19.5 | 19.5 | 19.6 | 19.5 | 0.2% | 0.0% | 0.2% | -0.1% | 0.1% |
| Cal04 | 27.3 | 29.1 | 28.1 | 28.5 | 1.8% | 6.4% | 2.7% | 4.3% | 4.5% |
| Cal05 | 42.9 | 46.8 | 46.9 | 42.6 | 5.4% | 9.1% | 9.1% | -0.9% | 5.8% |
| Cal06 | 74.2 | 74.9 | 80.3 | 70.3 | 6.6% | 1.0% | 8.2% | -5.2% | 1.3% |
| Cal07 | 136.7 | 138.3 | 132.0 | 128.8 | 3.6% | 1.2% | -3.4% | -5.8% | -2.7% |
| Cal08 | 261.7 | 244.9 | 278.9 | 285.2 | 8.0% | -6.4% | 6.6% | 9.0% | 3.0% |
| Cal09 | 511.7 | 506.1 | 468.1 | 520.9 | 5.5% | -1.1% | -8.5% | 1.8% | -2.6% |
| Cal10 | 1011.7 | 926.4 | 941.5 | 948.2 | 1.2% | -8.4% | -6.9% | -6.3% | -7.2% |
| Cal11 | 2011.7 | 1902.1 | 1970.6 | 1914.8 | 1.9% | -5.4% | -2.0% | -4.8% | -4.1% |

### Table 6. Comparison of the calibration curve generated in zero matrix to the standard addition calibration curve in native matrix (LTR-P) and its dilution series.

Comparison of the concentration values interpolated for each standard addition point in LTR plasma matrix and dilution series from the zero matrix averaged calibration curve to the concentration values extrapolated from the standard addition calibration curves and calculated concentration of diluted LTR plasma sample.

Linearity

Was assessed along with the method’s accuracy and precision in three separate days. The linear fit with 1/X^2^ weighting was applied to the response calculated as a ratio of ddhC peak area to the ^13^C_5_-ddhC SIL-IS peak area for each calibration concentration level. LLOQ was accepted from the tested linear range based on the 20% deviation of the back-calculated standard concentration with the relative standard deviation of the response value across all replicates of calibration curves not exceeding 20%.

Intra-day method accuracy and precision

Were assessed using three series of QC samples prepared at five concentration levels (QC1-QC5 described above). For acceptance, the QC were required to be within 15% (20% for QC1 = LLOQ QC) of their nominal concentration and 15% (20% for QC1) of %CV between the analysed replicates. Inter-day accuracy and precision were assessed in separate days with the total number of analysed QC samples (across day 1 and all additional days) of 13. The same acceptance criteria were used for the inter-day accuracy and precision assessment (Table 7).

|  | **Nominal concentration ng/ml** | **Intra-day accuracy % and precision (%CV)** | **Intra-day accuracy % and precision (%CV)** |
| --- | --- | --- | --- |
| **QC1** | 3.9 | 101.6 (3.1) | 99.0 (5.5) |
| **QC2** | 50 | 104.0 (3.1) | 100.7 (3.3) |
| **QC3** | 300 | 102.0 (4.1) | 98.1 (3.7) |
| **QC4** | 625 | 96.2 (4.0) | 99.1 (2.2) |
| **QC5** | 1200 | 101.8 (4.2) | 99.9 (3.3) |

### Table 7. Intra- (n=3) and Inter-day (n=13) % accuracy and precision as (%CV) for five QC concentrations and precision for LTR-P and LTR-S.

Carryover

Was assessed by injecting DB sample after ULOQ and QC5. Carryover acceptance criteria required that the MRM transitions of ddhC in the DB sample were less than 20% of LLOQ, and the MRM transitions of SIL-IS were less than 5% of the SIL-IS response detected in SB solution.

Selectivity

Was assessed using six individual sources of analyte-free matrix. These were generated using the described earlier SPE approach to remove endogenous ddhC from six healthy control plasma samples. The samples were prepared for selectivity analysis without spiking SIL-IS in. Absence of interfering components was accepted when the response was less than 20% of LLOQ for ddhC and less than 5% for the SIL-IS.

Matrix effect (ME)

Was assessed using six individual sources of analyte-free (zero) matrix prepared as described in Selectivity assessment using SPE to remove ddhC from six healthy control plasma samples. Each source of matrix was spiked with analyte and SIL-IS at concentration levels of 3.9 ng/mL (LLOQ QC) and 1200 ng/mL (high QC). The same concentrations were spiked into surrogate matrix (2% BSA in PBS). ME was calculated for each concentration as ME% = peak area zero matrix/peak area BSA. ME normalised by IS was calculated for each matrix source and each concentration as MEIS%=ME%(analyte)/ME%(IS), CV of MEIS% should be less than 15%. The results of ME assessment are shown in Table 8.

| **Spiked sample (each done in triplicate)** | **ME% (ddhC)** | **ME% (SIL-IS ddhC)** | **MEIS%=ME%(ddhC)**  **/ME% (SIL-IS ddhC)** | **Dev%** |
| --- | --- | --- | --- | --- |
| ME_HC1_3_9ngmL_01 | 129.8% | 126.0% | 103.0% | -3.0% |
| ME_HC1_3_9ngmL_02 | 121.2% | 124.4% | 97.5% | 2.5% |
| ME_HC1_3_9ngmL_03 | 118.3% | 124.2% | 95.3% | 4.7% |
| ME_HC2_3_9ngmL_01 | 119.7% | 120.0% | 99.8% | 0.2% |
| ME_HC2_3_9ngmL_02 | 121.8% | 130.5% | 93.3% | 6.7% |
| ME_HC2_3_9ngmL_03 | 108.9% | 125.1% | 87.1% | 12.9% |
| ME_HC3_3_9ngmL_01 | 117.9% | 131.5% | 89.6% | 10.4% |
| ME_HC3_3_9ngmL_02 | 121.7% | 120.9% | 100.6% | -0.6% |
| ME_HC3_3_9ngmL_03 | 117.1% | 124.2% | 94.3% | 5.7% |
| ME_HC4_3_9ngmL_01 | 115.0% | 128.2% | 89.7% | 10.3% |
| ME_HC4_3_9ngmL_02 | 122.4% | 124.9% | 98.0% | 2.0% |
| ME_HC4_3_9ngmL_03 | 117.6% | 117.4% | 100.2% | -0.2% |
| ME_HC5_3_9ngmL_01 | 106.8% | 111.1% | 96.1% | 3.9% |
| ME_HC5_3_9ngmL_02 | 109.6% | 119.8% | 91.5% | 8.5% |
| ME_HC5_3_9ngmL_03 | 108.1% | 114.5% | 94.5% | 5.5% |
| ME_HC6_3_9ngmL_01 | 109.3% | 114.2% | 95.7% | 4.3% |
| ME_HC6_3_9ngmL_02 | 108.1% | 112.3% | 96.3% | 3.7% |
| ME_HC6_3_9ngmL_03 | 85.2% | 85.4% | 99.7% | 0.3% |
|  | **ME% (ddhC)** | **ME% (SIL-IS ddhC)** | MEIS%=ME%(ddhC)  /ME% (SIL-IS ddhC) | Dev% |
| ME_HC1_1200ngmL_01 | 103.1% | 101.5% | 101.5% | -1.5% |
| ME_HC1_1200ngmL_02 | 97.7% | 86.1% | 113.4% | -13.4% |
| ME_HC1_1200ngmL_03 | 90.7% | 90.3% | 100.4% | -0.4% |
| ME_HC2_1200ngmL_01 | 93.9% | 91.2% | 102.9% | -2.9% |
| ME_HC2_1200ngmL_02 | 92.1% | 90.5% | 101.8% | -1.8% |
| ME_HC2_1200ngmL_03 | 91.0% | 90.2% | 100.8% | -0.8% |
| ME_HC3_1200ngmL_01 | 93.9% | 91.9% | 102.2% | -2.2% |
| ME_HC3_1200ngmL_02 | 91.7% | 92.0% | 99.7% | 0.3% |
| ME_HC3_1200ngmL_03 | 73.0% | 73.8% | 98.8% | 1.2% |
| ME_HC4_1200ngmL_01 | 97.1% | 95.6% | 101.6% | -1.6% |
| ME_HC4_1200ngmL_02 | 90.9% | 89.6% | 101.4% | -1.4% |
| ME_HC4_1200ngmL_03 | 67.9% | 67.3% | 100.8% | -0.8% |
| ME_HC5_1200ngmL_01 | 88.9% | 90.2% | 98.5% | 1.5% |
| ME_HC5_1200ngmL_02 | 92.8% | 89.3% | 104.0% | -4.0% |
| ME_HC5_1200ngmL_03 | 85.3% | 86.0% | 99.2% | 0.8% |
| ME_HC6_1200ngmL_01 | 94.4% | 86.9% | 108.5% | -8.5% |
| ME_HC6_1200ngmL_02 | 90.7% | 96.8% | 93.6% | 6.4% |
| ME_HC6_1200ngmL_03 | 90.8% | 95.7% | 94.9% | 5.1% |

### Table 8. ME assessment results.

Matrix Effect (ME %) was measured using six individual ddhC-free (zero) plasma samples, obtained from healthy controls, that were treated with SPE to remove endogenous ddhC and spiked with known concentration of ddhC corresponding to LLOQ QC (3.9 ng/mL) and high QC (1200 ng/mL) and SIL-IS ^13^C_5_-ddhC. Each spiked QC sample was measured in triplicate. Each peak area of ddhC measured in the zero matrix were referenced to the same concentrations of ddhC standard spiked in surrogate matrix (2% BSA in PBS).

Stability

Was assessed using LLOQ QC and high QC prepared in three replicates for both concentration levels for each test. Quantification of the replicates of the QC samples and subsequent stability assessment was made following sample storage at 4°C (in the autosampler) for 24 and 72 hours, at -80°C for 24 hours, 72 hours, and 14 days, and at -20°C for 14 days. The acceptance criteria required accuracy and precision of the determined concentrations of LLOQ QC and high QC after each storage condition to be within 20% and 15%, respectively (Table 9).

| **Stability condition** | **Nominal Concentration** | **Average Observed Concentration (n=3)** | **Accuracy** | **% CV** |
| --- | --- | --- | --- | --- |
|  | **[ng/mL]** | **[ng/mL]** | **%** | % |
| Stability Reference (T0) | 3.9 | 4.6 | 118.8 | 21.0 |
| Stability Reference (T0) | 1200 | 1237.0 | 103.1 | 3.0 |
| Stability 24 hours, 4°C | 3.9 | 3.3 | 84.6 | 5.0 |
| Stability 24 hours, 4°C | 1200 | 1157.5 | 96.5 | 1.7 |
| Stability 72 hours, 4°C | 3.9 | 3.8 | 98.3 | 5.4 |
| Stability 72 hours, 4°C | 1200 | 1238.5 | 103.2 | 0.4 |
| Stability 24 hours, 80°C | 3.9 | 3.3 | 84.6 | 2.5 |
| Stability 24 hours, 80°C | 1200 | 1205.5 | 100.5 | 1.8 |
| Stability 72 hours, 80°C | 3.9 | 4.0 | 102.6 | 4.1 |
| Stability 72 hours, 80°C | 1200 | 1201.9 | 100.2 | 1.5 |
| Stability 14 days, 80°C | 3.9 | 4.5 | 116.2 | 1.0 |
| Stability 14 days, 80°C | 1200 | 1289.6 | 107.5 | 4.1 |
| Stability 14 days, 20°C | 3.9 | 4.5 | 114.5 | 3.8 |
| Stability 14 days, 20°C | 1200 | 1261.5 | 105.1 | 1.4 |

### Table 9. Stability assessment results.

Accuracy and precision of the ddhC quantification were assessed at two different concentration levels after storing the prepared solutions in different temperature conditions for a different duration of storage.

Dilution linearity

Was assessed by preparing a calibration solution at concentration of 2xULOQ (4000 ng/mL) and diluted 4 and 16 times using the diluent matching the composition of the single blank sample (SB). Each dilution was prepared in five replicates which were quantified using ddhC calibration curve. The acceptance criteria required accuracy and precision of the determined concentrations of diluted 2xULOQ to be within 15% (Table 10).

| **Dilution Integrity x16 and x4 (4000mg/mL)** | **Nominal Concentration** | **Observed Concentrations** | **Accuracy** | **Average observed concentration** | **% CV** |
| --- | --- | --- | --- | --- | --- |
|  | **[ng/mL]** | **[ng/mL]** | **%** | **[ng/mL]** |  |
| DIx16_01 | 250 | 243.7 | 97.5 |  |  |
| DIx16_02 | 250 | 258 | 103.2 |  |  |
| DIx16_03 | 250 | 249.9 | 100.0 |  |  |
| DIx16_04 | 250 | 252.5 | 101.0 |  |  |
| DIx16_05 | 250 | 261.8 | 104.7 | 253.2 | 2.5 |
| DIx4_01 | 1000 | 979.5 | 98.0 |  |  |
| DIx4_02 | 1000 | 969.3 | 96.9 |  |  |
| DIx4_03 | 1000 | 965.2 | 96.5 |  |  |
| DIx4_04 | 1000 | 968.6 | 96.9 |  |  |
| DIx4_05 | 1000 | 967.5 | 96.8 | 970 | 0.5 |

### Table 10. Dilution integrity assessment results.

### Data processing

Raw UHPLC-ESI-MS/MS spectral data were processed using the TargetLynx application package within MassLynx (v4.1) software (Waters Corporation). Microsoft Excel was used for the validation data assessment.

## References

1. Killingley, B.*, et al.* Safety, tolerability and viral kinetics during SARS-CoV-2 human challenge in young adults. *Nature Medicine* **28**, 1031-1041 (2022).

2. Temple, D.S.*, et al.* Wearable Sensor-Based Detection of Influenza in Presymptomatic and Asymptomatic Individuals. *J Infect Dis* **227**, 864-872 (2023).

3. Jackson, G.G., Dowling, H.F., Spiesman, I.G. & Boand, A.V. Transmission of the common cold to volunteers under controlled conditions. I. The common cold as a clinical entity. *AMA Arch Intern Med* **101**, 267-278 (1958).

4. Buang, N.*, et al.* Type I interferons affect the metabolic fitness of CD8+ T cells from patients with systemic lupus erythematosus. *Nature Communications* **12**, 1980 (2021).

5. Uppala, R.*, et al.* HERC6 regulates STING activity in a sex-biased manner through modulation of LATS2/VGLL3 Hippo signaling. *iScience* **27**(2024).

6. Busse, D.C.*, et al.* Interferon-Induced Protein 44 and Interferon-Induced Protein 44-Like Restrict Replication of Respiratory Syncytial Virus. *Journal of Virology* **94**, 10.1128/jvi.00297-00220 (2020).

7. Bosquillon de Jarcy, L.*, et al.* 90K/LGALS3BP expression is upregulated in COVID-19 but may not restrict SARS-CoV-2 infection. *Clinical and Experimental Medicine* **23**, 3689-3700 (2023).

8. Mar, K.B.*, et al.* LY6E mediates an evolutionarily conserved enhancement of virus infection by targeting a late entry step. *Nature Communications* **9**, 3603 (2018).

9. Ding, H.*, et al.* Membrane Protein OTOF Is a Type I Interferon-Induced Entry Inhibitor of HIV-1 in Macrophages. *mBio* **13**, e01738-01722 (2022).

10. He, X.*, et al.* RTP4 inhibits IFN-I response and enhances experimental cerebral malaria and neuropathology. *Proceedings of the National Academy of Sciences* **117**, 19465-19474 (2020).

11. Kim, N.*, et al.* Interferon-inducible protein SCOTIN interferes with HCV replication through the autolysosomal degradation of NS5A. *Nature Communications* **7**, 10631 (2016).

12. Zheng, Q.*, et al.* Siglec1 suppresses antiviral innate immune response by inducing TBK1 degradation via the ubiquitin ligase TRIM27. *Cell Research* **25**, 1121-1136 (2015).

13. Chen, M.*, et al.* SPATS2L is a positive feedback regulator of the type I interferon signaling pathway and plays a vital role in lupus. *Acta Biochimica et Biophysica Sinica* **56**, 1659-1672 (2024).

14. Wang, K.*, et al.* Interferon-stimulated TRIM69 interrupts dengue virus replication by ubiquitinating viral nonstructural protein 3. *PLOS Pathogens* **14**, e1007287 (2018).

15. Jeong, S.-I.*, et al.* XAF1 forms a positive feedback loop with IRF-1 to drive apoptotic stress response and suppress tumorigenesis. *Cell Death & Disease* **9**, 806 (2018).

16. Lewis, M.*, et al.* An open platform for large scale LC-MS-based metabolomics. *preprint ChemRxiv* (2022).

17. ICH M10 on bioanalytical method validation guideline. Available at: <https://www.ema.europa.eu/en/documents/scientific-guideline/ich-guideline-m10-bioanalytical-method-validation-step-5_en.pdf>.

18. M10 Bioanalytical Method Validation and Study Sample Analysis. Available at: <https://www.fda.gov/media/162903/download>. .

19. Fernandez-Metzler, C.*, et al.* Biomarker Assay Validation by Mass Spectrometry. *AAPS J* **24**, 66 (2022).
